# Water-Mediated Dissemination and Detection of Antibiotic Resistance Across Livestock, Agri-Food, and Aquaculture Systems

**DOI:** 10.3390/mi16080934

**Published:** 2025-08-13

**Authors:** Debora Pinamonti, Jasmina Vidic, Michela Maifreni, Alessia Cossettini, Vincent Leguillier, Marisa Manzano

**Affiliations:** 1 Department of Agricultural, Food, Environmental and Animal Science, University of Udine, 33100 Udine, Italy; pinamonti.debora@spes.uniud.it (D.P.); michela.maifreni@uniud.it (M.M.); cossettini.alessia@spes.uniud.it (A.C.); 2 Université Paris-Saclay, INRAE, AgroParisTech, Micalis Institute, 78350 Jouy en Josas, France; jasmina.vidic@inrae.fr (J.V.); vincent.leguillier@inrae.fr (V.L.)

**Keywords:** antibiotic-resistant bacteria, antibiotics, One Health, alternative to antibiotics, surveillance, detection

## Abstract

Currently antibiotic resistance is one of the biggest threats to human and animal health. Its spread has been increasing around the world since the mid-20th century; thus, prevention and understanding of the causes are needed. The issue of antibiotic resistance is often attributed to the healthcare sector, yet numerous other sectors, such as the environment and the agri-food sector, also contribute to the spread of resistant bacteria. The presence of pharmaceutical residues and bacterial contaminants in sewage, landfills, food raw materials, and food industries promotes the selection and proliferation of resistant bacteria, including pathogenic strains that pose a threat to human and animal health. Water quality must be kept under control because microorganisms resistant to antibiotics can find suitable conditions to live, multiply, and be transported. This review focuses on recent findings on the role of water as a transmission route for antibiotic resistance across the livestock, agri-food, and aquaculture sectors. We mapped the full pathway of resistant bacteria, from environmental and raw food sources to the end consumer, and outlined future strategies for monitoring and control of antibiotic resistance.

## 1. Introduction

Antibiotic resistance (AR) threatens the effectiveness of therapies against an ever-increasing range of infections caused by bacteria and is responsible for severe hazards to public health [1]. In 2019 alone, antimicrobial resistance, including AR, was directly responsible for 1.27 million deaths worldwide [2], a staggering picture that could escalate to 10 million people’s deaths annually by 2050 if no decisive action is taken [3].

Bacteria adapt rapidly to drugs, developing resistance mechanisms that render commonly used drugs, such as penicillin, quinolones, cephalosporins, macrolides, and sulphonamides, ineffective [4]. This evolutionary adaptation has led to the emergence of multidrug resistant “superbugs”, whose treatment is currently one of the main challenges to both human and veterinary medicine. The ESKAPE pathogens (*Enterococcus faecium*, *Staphylococcus aureus*, *Klebsiella pneumoniae*, *Acinetobacter baumannii*, *Pseudomonas aeruginosa*, and *Enterobacter* species) exemplify this threat. This acronym highlights the microorganisms’ ability to select, transmit, and replicate resistance determinants, enabling them to evade antibiotic treatments [5,6]. Initially identified as a group of nosocomial pathogens, these microorganisms are not only responsible for numerous diseases linked to AR in hospital settings, but they are also increasingly detected in environmental reservoirs [7]. Moreover, zoonotic bacteria such as *Salmonella*, *Escherichia*, *Campylobacter*, and *Staphylococcus* spp., common bacteria in agricultural, aquaculture and food-processing environments, can transmit AR determinants reaching humans via water, food and direct contact with animals [8]. The water systems act as both reservoir and conduit for residues of antibiotics, fecal contaminants and AR bacteria, favouring the horizontal gene transfer in microbial communities [9,10]. For instance, *E. coli*, a fecal contamination indicator bacterium, can persist in drinking water for up to 12 weeks [11]. The European Union (EU) Directive 2020/2184 imposes rigorous monitoring of such contaminants in water intended for human consumption and food processing [12]. To ensure the safe reuse of water, including potable purposes, advanced treatment technologies are required to remove pollutants of emerging concern, such as antibiotics and antibiotic resistance genes (ARGs) [13]. Low- and middle-income countries (LMICs) are severely vulnerable to AR, largely due to poor sanitation systems, weak enforcement and monitoring infrastructure, and antibiotic misuse. It is therefore crucial to take into account regional variations in the diversity and abundance of ARGs, as well as the potential for improved hygiene to limit the global burden of AR [14,15]

**Figure 1:**
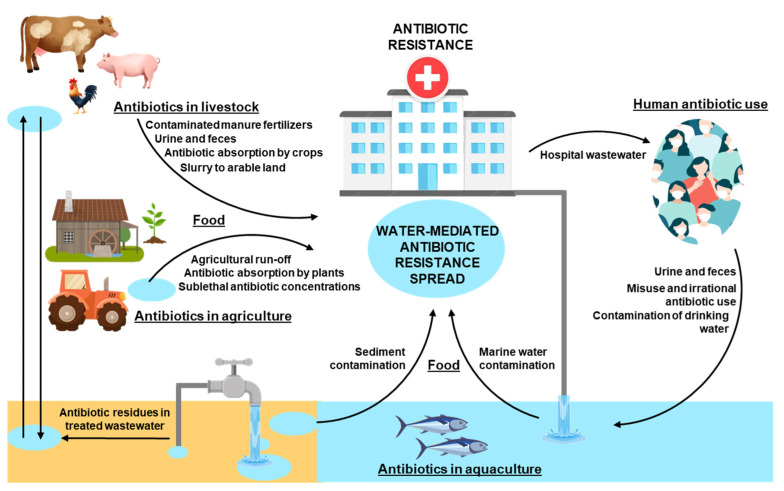
Water as one of the main global transmission routes of antibiotic resistance. Scheme representing the transmission of antibiotic resistance between livestock, agriculture, aquaculture and humans.

Given the interconnection of water systems with agricultural and food production chains (Figure 1), the dissemination of AR via water is a critical but often unnoticed public health concern.

## 2. AR Bacteria in the Environment

### 2.1. Occurrence of AR Bacteria in Livestock Waste

Livestock farming is a significant contributor to the environmental dissemination of AR bacteria. According to the 2022 EFSA report for the period 2019–2020, *Salmonella* spp., *E. coli*, *Campylobacter* spp., and *S. aureus* were identified as priority AR pathogens among European livestock populations, specifically in poultry and pigs under one year of age [8]. Several other reports emphasized the growing concern over the presence of resistant strains of *E. coli*, *S. aureus* and *A. baumannii* in various livestock environments [16,17,18,19,20]. These AR profiles are frequently associated with antibiotics commonly used in both veterinary and human medicine, such as penicillins, tetracyclines, macrolides, sulfonamides, fluoroquinolones and quinolones, and lincosamides [21]. Improperly treated livestock waste, containing both resistant bacteria and residual biologically active drug compounds, can contaminate surrounding water bodies, leading to the accumulation of AR bacteria in the environment and acting as a source of selective pressure on aquatic bacterial populations [22,23]. Analyses performed on pig farms detected the presence of *Salmonella* strains resistant to tetracycline, sulfisoxazole, and streptomycin, not only in animal waste but also in adjacent water sources, indicating a shared resistance profile likely due to waste runoff [24]. Likewise, *Salmonella* strains resistant to sulphamethoxazole, chloramphenicol, and ampicillin were identified in both pig wastes and on-farm stream waters, suggesting surface water as a vehicle for the off-farm spread of AR bacteria [25]. In rural Chinese water systems affected by nearby livestock production, *E. coli* isolates exhibited profiles of multidrug resistance to sulphamethoxazole/trimethoprim, gentamycin, ampicillin, tetracycline and chlortetracycline, suggesting a possible cross-selection and bidirectional flow of AR between water and the animals [23]. Complementary findings [26] reported the presence of tetracyclines, fluoroquinolones, and sulfonamides in livestock feces in the same area, reinforcing the link between antibiotic use and environmental persistence. Water contamination with *A. baumannii*, in particular strains resistant to tetracycline and sulphamethoxazole/trimethoprim, was found at high rates in wastewater channels and rivers adjacent to livestock farms, suggesting these aquatic environments as critical sources or recipients strongly related to AR [20]. Additionally, Hsu et al. [27] confirmed the widespread resistance to sulfonamides in water bodies impacted by intensive animal farming. Multidrug-resistant *Campylobacter* spp., especially *C. jejuni* and *C. coli*, were isolated from samples within poultry farms, such as cloacal swabs, tap water, attendants’ hand-rinsed water and whole carcasses. These isolates showed resistance to amoxicillin, azithromycin, ciprofloxacin, erythromycin, gentamycin, norfloxacin, streptomycin, and tetracycline, reflecting extensive environmental dissemination [28].

As a result, these findings underscore how livestock waste acts as a hotspot for AR emergence and environmental release. This scenario poses significant risks not only to the environment but also to the agricultural sector, food chain, and public health. As shown in Table 1, multiple studies reported the high prevalence of AR bacteria in livestock, underlining the urgent need to re-evaluate livestock waste management practices to mitigate downstream contamination.

### 2.2. Occurrence of AR Bacteria in Agricultural Waste

Sublethal antibiotic concentrations in wastewater, even after treatments, have been labelled as a potential contributor to AR development and spread, with the possibility of reaching the human population through foodborne transmission [29].

Water recovered from treated wastewater is increasingly used for irrigation purposes, especially in water-scarce regions; however, this water may pose a risk for resistance transmission onto fresh agricultural products through absorption by crops. Hu et al. [30] demonstrated the presence of typical veterinary antibiotics, specifically tetracyclines, sulphonamides, and quinolones, in manure, soil, groundwater, and vegetables, confirming the transfer of these compounds through food production. Despite wastewater treatments aimed at removing external substances and contaminants, AR bacteria can persist and be disseminated into the surrounding water bodies, distributed on fields, and transported via precipitation and overland flow. This transport mechanism was investigated at various points of agricultural production to assess the risk of transportation of veterinary pharmaceutical residues to edible plant products [31]. For instance, oxytetracycline was detected in sediments and soil fertilized by pig slurry [32] and was also found in overland flow following land application of slurry to arable land. Similarly, Sun et al. [33] confirmed the presence of ionophore antibiotics, in particular monensin, salinomycin, and narasin, in poultry litter–soil–water systems, highlighting their environmental persistence and transport. In addition, biofilm formation in aqueous settings enhances AR in bacteria, hindering penetration of antibiotics and activating adaptive stress responses that contribute to microbial survival mechanisms [34]. The microorganism’s ability to communicate through small signalling molecules, known as autoinducers, is responsible for the sharing of information that enables the bacterium to express genes, including those of its virulence factors, amplifying the capacity of AR bacteria to persist in agricultural ecosystems.

A connection between antibiotics dissolved in water and microorganisms is hypothesized to explain the development of AR bacteria in agricultural raw materials at the basis of human food nutrition. *Salmonella* spp., isolated in swine manure used to fertilize lands, exhibited resistance to streptomycin, sulfisoxazole, and tetracycline and was capable of surviving in soil, with the consequent possibility of reaching watercourses. It persisted in the soil, and it was resistant to streptomycin, sulfisoxazole, and tetracycline [35]. Similarly, Agostinho Avanci et al. [36] found *E. coli* resistant to tetracycline, gentamicin, cefotaxime, nitrofurantoin, trimethoprim-sulfamethoxazole, and ampicillin in avian-based organic fertilizer, while strains of *E. coli* O157:H7 resistant to quinolone were detected in irrigation water by Abakpa et al. [37], further underlining the role of water as a transmission medium. Environmental sources, in particular irrigation water, were linked to AR found in vegetal products, as reported by Hudson et al. [38], reinforcing the notion that water reuse significantly favours AR and multidrug-resistance spread to fresh products and, ultimately, to human consumers.

In conclusion, agricultural environments represent critical interfaces for the transfer and persistence of AR bacteria, with water bodies serving as crucial reservoirs and vectors. Their ability to transport resistant microorganisms through soils, crops, and surface runoff highlights the urgency of integrated monitoring strategies to safeguard food safety and mitigate AR dissemination routes. Table 2 summarizes the recent findings regarding the presence of AR in agricultural settings.

### 2.3. Occurrence of AR Bacteria in Aquaculture Waste

As global food demand rises, European aquaculture is expected to reach 4 million tons by 2030 to meet the increasing population’s needs. The land-based production is estimated to be insufficient to satisfy this request, leading to the need for food from aquaculture production. To sustain intensive fish farming and prevent disease outbreaks, antibiotics are sometimes used, contributing to the emergence and spread of AR bacteria in aquatic environments [39,40]. Aquaculture was implicated in the selection and transmission of resistance genes, often facilitated by horizontal gene transfer by plasmids [41,42]. Sulphonamide, tetracycline, quinolone, and erythromycin are just some of the common drugs used in fish farming, and the ARGs against these antibiotics can easily spread among bacteria in aquatic microbial communities [43,44]. According to a recent scientific report on AR bacteria [45], fish species farmed in Europe harboured AR microorganisms resistant to ampicillin, enrofloxacin, florfenicol, flumequine, oxolinic acid, sulfonamide–trimethoprim and oxytetracycline, antibiotics of clinical relevance in both veterinary and human medicine. Extended-Spectrum Beta-Lactamase (ESBL)-producing *E. coli* were isolated from aquaculture settings, confirming the sector’s role as a reservoir for clinically significant resistance traits [46].

The aquatic environment not only holds resistant strains locally but also serves as a medium for their dispersal over long distances. Shah et al. [47] discovered resistant bacteria in a site 8 km away from salmon farming areas, likely the result of transport by water currents. Resistances to tetracycline, trimethoprim, sulfamethizole, amoxicillin, and streptomycin were identified in marine bacteria. Numerous AR bacteria isolated from sediments of aquaculture watercourses showed resistance to florfenicol, sulfonamide, streptomycin, aminoglycoside, tetracycline, chloramphenicol, and trimethoprim [48,49].

The global public health implications are considerable. AR is expanded to many parts of the world concerning aquaculture, and water acts as a global reservoir of both clinically relevant and potentially novel ARGs [50]. It was estimated that in 2019 alone, over 50,000 deaths were attributed to infections caused by AR *E. coli* and *K. pneumoniae* resistant to fluoroquinolones, carbapenem and β-lactamase inhibitor combinations [2]. Alarmingly, farmed fish and aquaculture water were found contaminated with AR *E. coli* and *K. pneumoniae* [51]. These findings underscore that aquatic settings act as contributors to AR diffusion. Infections in human individuals may, in some cases, derive from exposure to aquaculture products and their associated aquatic environments, where selective pressures favour the proliferation of AR bacteria. This raises concerns about the contamination of aquacultural products intended for human consumption by AR bacteria present in water sources. Table 3 summarizes some recent data related to AR in aquaculture.

## 3. Occurrence of AR Bacteria in Retail Foods

Retail food products represent a direct interface between environmental contamination and human consumer exposure. Food processing, through conventional and innovative technologies, is developed to guarantee food safety [52], but cross-contamination and post-treatment recontamination can occur throughout the food chain and water, widely used in food processing, washing, and as an ingredient, may act as a vector of AR to food and consumers. Resistant bacteria were detected even in heat-treated or dried foods, suggesting post-treatment contamination during storage or distribution [53,54,55]. Endospore-forming bacteria, such as *Bacillus cereus* or *Clostridium perfringens*, in their sporulated form, may survive most of the procedures used by the food industry. Bacterial spores can remain dormant for an extensive time and then germinate and regrow under more favourable conditions. Interestingly, *B. cereus* strains isolated from pasteurized milk exhibited resistance to ampicillin, lincomycin, erythromycin, and tetracycline, raising concerns about potential horizontal gene transfer from non-pathogenic to pathogenic strains [56]. Several studies report the presence of multidrug-resistant bacteria in ready-to-eat foods, with contamination often traced back to irrigation water, wash water, or surfaces in processing environments. *Acinetobacter* spp., *E. coli*, and *Salmonella* have been isolated from fresh produce, highlighting water’s pivotal role in transferring resistance from agricultural or aquacultural systems into the human food chain. Interestingly, indirect exposure of consumers to resistant *E. coli* was detected: *E. coli* responsible for human diseases in vegetarian individuals were found to be very similar to resistant *E. coli* poultry strains, suggesting environmental and indirect foodborne routes of transmission, rather than direct meat consumption alone [38]. This supports the concept that the environment, not only raw materials, is a critical reservoir of resistance: a direct relationship between food consumption and disease was not always identified, leading to suppose that the cause of the spread of resistance was not always the raw material, but the environment and the contaminated water from the livestock and agricultural sectors previously discussed.

Another relevant concern is the use of bacteria in food biocontrol. Lactic acid bacteria and probiotics must respond to the requirement of being in the Qualified presumption of safety (QPS) list, which requires the absence of acquired ARGs to antibiotics of medical relevance [57]. However, evidence suggests that some technological strains may harbour mobile resistance genes, posing a risk of horizontal gene transfer during food production or in the gut microbiota of consumers [58]. The discussion can be extended to all bacteria, in line with information that confirms that the bacteria included in the QPS list are not a source of risk, while other microorganisms must be considered as potential supporters of AR, especially from the aquatic environment, which is a promoter of horizontal gene transfer and must be carefully evaluated. In addition, wastewater generated from food production represents AR hotspots and an important source of environmental pollution. Studies on wastewater treatment plants isolated multidrug-resistant *E. coli* capable of surviving treatment, colonizing the sewer system, and forming strong biofilms, which serve as reservoirs and vehicles for the persistence and dissemination of ARGs [59,60]. Water and the food production environment include biofilms with a high abundance of opportunistic pathogens, able to increase horizontal gene transfer and promote AR spread on equipment, food contact surfaces, and areas that are difficult to clean and disinfect, such as dead ends, edges, joints, and seals [61,62]. Taken together, these findings highlight the complex interplay between water, food, and AR bacteria, where the water cycle acts as a continuous driver of resistance transmission across sectors. The strong interconnection between AR bacteria and water as a means of transport around all environments is illustrated in Figure 1.

## 4. Prevention and Monitoring of AR Bacteria

The World Health Organization (WHO) has introduced global plans to combat AR, such as “The Global Action Plan” and “Global Antimicrobial Resistance and Use Surveillance System” based on the One Health concept of the inseparable link between human, animal, and environmental health [63,64]. In addition to the WHO-recommended strategies to tackle AR, the development of antibiotic alternatives and improvements in target measures through strong international cooperation are essential to minimize the development of AR.

The rapid emergence of AR pathogens has prompted the search for alternative antibacterial agents, such as antimicrobial peptides (AMPs), bacteriophages, and antibacterial nanoparticles (NPs). AMPs have natural antibiotic properties with a broad spectrum of activity through their interaction with the bacterial cell membrane. The interaction between AMP and bacterial cells is guided by electrostatic forces between peptides’ positive charge and the negatively charged membrane structures [65]. This interaction with bacterial membranes instead of intracellular targets reduces the development of microbial resistance. One promising class of AMPs are lipopeptides that contain a hydrophilic peptide attached to a fatty acyl side chain, which promotes insertion and drug clustering in the biological membranes. Daptomycin and vancomycin are examples of lipopeptide antibiotics. They are used as a last resort antibiotic against multidrug-resistant enterococci and staphylococci [66]. Other, AMP-based effective therapies have been developed against the main AR pathogens, such as histatin 5, a salivary peptide produced in humans and higher primates, that has bactericidal activity against *S. aureus* and *A. baumannii* [67].

Bacteriophages (phages) are naturally occurring viruses that infect and kill bacteria. They adhere to the bacterial cell through tail fibres, transfer the nucleic acid inside the cell, make many copies of themselves, and lastly, the newly assembled phages cause cell membrane lysis, killing the bacterium. Treatment of infections caused by bacteria using this alternative solution is possible thanks to careful evaluation of the phages for their effectiveness against a specific isolated pathogen by using a mixture of phages, called a phage cocktail [68]. Several studies carried out in vitro proved phages to be effective as antibacterial agents against biofilm and bacteria. For example, some phages isolated from wastewater had lytic activity and were able to reduce the bacterial load of multidrug-resistant *S. aureus* [69,70]. Nevertheless, the full success of novel therapeutic strategies beyond the clinical perspective will include education, accessibility, and cultural and economic growth.

Metal and metal oxide nanoparticles in aqueous solutions may produce Reactive Oxygen Species (ROS) and/or release toxic metal ions, which endow them with antibacterial activity [71]. Nano-sized silver (Ag NPs), zinc oxide (ZnO NPs), and titanium oxide (TiO NPs) are employed in aquaculture, livestock, and poultry farms to combat pathogens. Their application contributes to an increase in economic value by preventing diseases. Due to their small sizes, nanoparticles may penetrate bacterial biofilms and efficiently eradicate them [72]. In aquaculture, antibacterial inorganic nanoparticles are used for water treatment to improve production. The combination of 0.1 mg/L of Ag NPs and 2 mg/L ZnO NPs was identified as optimal for effective bacterial removal [73]. When applied at low quantities, the adverse effects of these NPs can be limited. In surface waters, Ag NPs were reported in a concentration between 0.06 and 16 ng/L, while ZnO NPs were between 1.7 and 21 μg/L [74]. However, according to the WHO, silver concentrations in environmental water were measured at higher levels, ranging from 0.2 to 0.3 mg/L. The accumulation of antibacterial nanoparticles in water and soil may pose novel concerns because they can kill environmental non-target bacteria. These NPs can transform and accumulate in sludge or be discharged in effluents, potentially impacting aquatic ecosystems and causing ecotoxicological risks. Excessive production of ROS by ZnO NPs can induce toxic effects, including oxidative damage to DNA, lipid peroxidation, and protein denaturation. Similarly, Ag NPs can reduce the availability of organic matter, increase redox potential, and damage protective bacterial structures, further compromising microbial activity. For drinking water, the maximum allowable level of zinc was established by the Environmental Protection Agency (EPA) at 5 mg/L and that of silver was up to 0.1 mg/L [75]. The biodegradation of engineered nanomaterials should be considered at the early stages of their development. In agriculture, NPs can alter the physicochemical properties of the rhizosphere, leading to both positive and negative effects on the soil microbiota that support agricultural productivity [76]. Furthermore, the potential bioaccumulation of nanoparticles through the food chain from water and plants was identified as a possible risk to human health [77]. However, recent studies emphasize the need for further investigation, as current evidence remains limited and not yet fully understood [78]. This concern is reinforced by recent findings which show bioaccumulation of nanoparticles in certain fish species. Nevertheless, the associated health risk from consuming these aquatic products appears to be low due to limited intestinal absorption. Still, long-term health implications require more in-depth study [79].

Another critical point to prevent the spread of AR relies on the rapid detection of ARGs and resistance determinants. Considering various mechanisms of resistance due to the large-scale application of antibiotics, the monitoring of specific ARGs necessitates a quick protocol or tool to avoid the long time required by classical microbiological antibiotic susceptibility testing [80,81]. PCR protocols have been developed for the most prevalent and clinically relevant ARGs, such as the methicillin- (*mecA*) and vancomycin-resistance (*vanA*) genes in *S. aureus*; genes encoding for determinants responsible for carbapenem and β-lactams resistance (*CTXM*, *OXA-48*, *blaKPC*, *blaNDM-1*) in *K. pneumoniae*, carbapenem and β-lactams resistance (*blaIMP*, *blaOXA51*) in *A. baumannii*, and carbapenem and β-lactams resistance (VIM, OXA, metallo-β-lactamase-1) in *Enterobacter* spp.; and *E. coli* ARGs coding for CTX-M-1, TEM-116, TEM-52 and SHV12 β- lactamases [82,83,84]. However, the absence of a particular gene cannot guarantee the susceptibility of the strain to a particular antibiotic, while negative PCR results do not necessarily mean no resistance because there are several genes responsible for the same resistance, and the gene can mutate to encode an inactive protein. In addition, the limit of detection of PCR is about 50 CFU/mL. It is estimated that 10% of cases with low bacterial loads are not detected using PCR. Other limitations of molecular tests to detect ARGs come from specific resistance mechanisms. For example, daptomycin is active against Gram-positive but not against Gram-negative bacteria due to a different cytoplasmic membrane composition, especially the anionic phospholipid proportion [85]. Moreover, the intrinsic resistance of Gram-negative bacteria to many antibiotics is caused by the inability of drugs to cross the outer membrane and the action of efflux transporters. A different membrane composition or modification of efflux transporters can decrease/increase cell permeability and sensitivity to specific antibiotics. A PCR method cannot be used for the understanding of such resistance mechanisms.

In France, two emerging extensively drug-resistant bacteria are CPE and Glycopeptide-Resistant *Enterococci* (GRE). Although still sporadic in France, CPE and GRE prevalence is worrying in countries such as India, Italy, and Greece [63,64]. To limit their cross-transmission and prevent an endemic situation, there is an urgent need to rapidly detect carriers of that resistance. Currently, some rapid kits are being developed and commercialized, but they show limitations. The rapid lateral flow test NG-Test Carba 5 (NG Biotech, Guipry-Messac, France) has a limit of detection of 10^5^ CFU/mL [86], while biochemical tests, such as RAPIDEC CARBA NP (BioMérieux, Marcy-l’Etoile, France), have a limit of detection of more than 10^8^ bacteria [87]. Both tests provide results from 30 min to 2 h. Low sensitivity of tests is a particular problem when environmental, food, and water samples have to be examined because they typically contain a very low bacterial load between 1 and 10 CFU/mL. Consequently, rapid tests are usually applied after an enrichment step or magnetic bacterial preconcentration [88].

**Figure 2:**
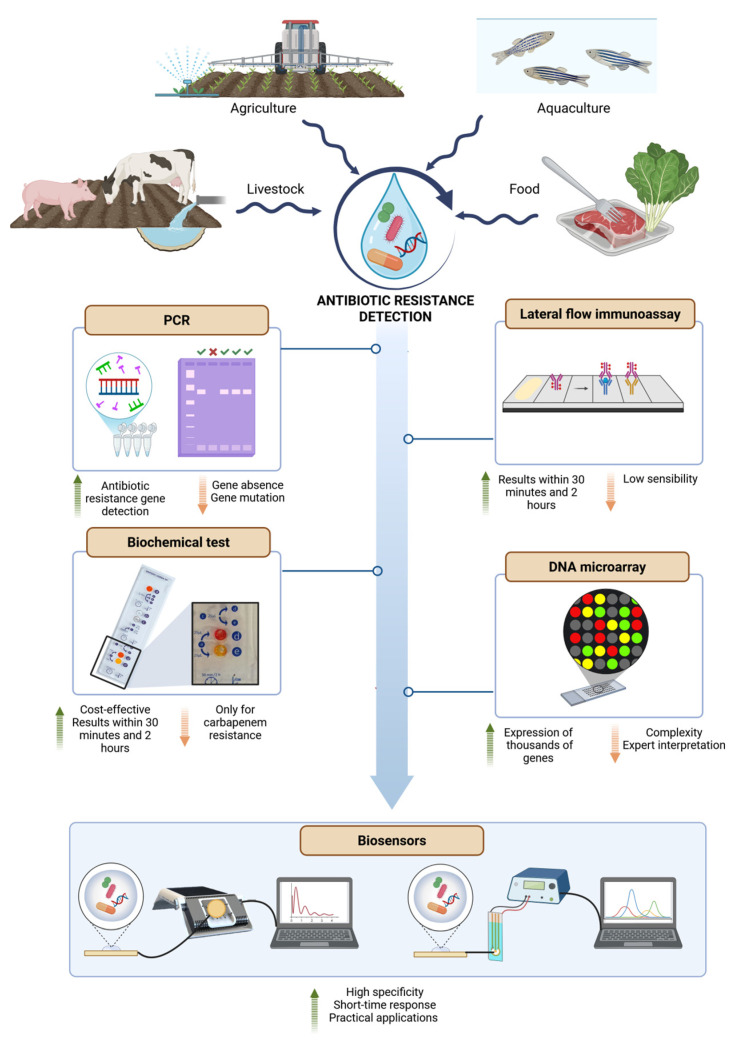
Summary of the main analytical tools used in aqueous environments closely related to raw food materials and the finished food product to detect the presence of antibiotic resistance. Water in livestock farming, agriculture, aquaculture, and food matrices can act as a carrier of antibiotics, resistant bacteria, and antibiotic resistance genes. These contaminants are identified using various analytical techniques, including PCR, lateral flow immunoassays, biochemical tests, DNA microarrays, and biosensors. PCR provides information on the presence of resistance genes but cannot detect gene absence or mutations. Lateral flow immunoassays deliver rapid results (30 min to 2 h) but are limited by low sensitivity. Biochemical tests are cost-effective and provide fast results (30 min to 2 h), yet they are typically limited to specific resistances (e.g., carbapenem resistance). DNA microarrays offer broad detection of thousands of genes; however, the method is complex and requires trained personnel. Biosensors represent the most promising tool, combining high specificity, rapid response time, and practical applicability. Advantages and disadvantages of each method are indicated with green and orange arrows, respectively.

DNA microarray analysis has opened up new interesting investigative potentials to simultaneously search for a large number of genes that can be involved in the spread of AR. The screening of a large number of genes provides molecular information on sequences and single-point mutations that can make the antibiotic inefficient. DNA microarrays reveal the expression of thousands of genes (active and repressed genes), analyzing mixed environmental samples, therefore giving the possibility to investigate the transfer of ARGs from water to food and vice versa, considering external factors, such as climate change and production practices [89]. Finally, whole genome sequencing (WGS) of bacteria has shown potential for epidemiological surveillance and infection control, and whole metagenome sequencing (WMS) allows for the culture-independent analysis of complex microbial communities. Both these omics technologies can assist in the tracking of ARGs, providing the necessary data for the implementation of risk assessments and the identification of hotspots and routes of AR transmission across the food chain through water. The current WGS technology provides a more comprehensive picture of all ARGs that may be present in an isolate and offers the possibility to rapidly add new target sequences to the analysis database, as well as the ability to perform fast analysis on already sequenced isolates. The main challenges are obtaining complete databases containing the relevant DNA sequence targets and applying appropriate bioinformatic methodologies to accurately extract the information from WGS technology [90]. Alternatively, WMS involves the fragmentation and subsequent sequencing and assembly of total genomic DNA isolated from a sample, making it possible to gain information on its entire gene content. Some examples of WMS applications in the food science field are the detection of foodborne pathogens in food, the investigation of the transmission of microorganisms through livestock and agricultural settings, and the identification of changes in microbial populations. There are several functions, and the analysis of the AR is closely related to this technology to guarantee environmental and food safety [91]. WMS has also been demonstrated to be useful when considering the serious problem of the horizontal gene transfer of ARGs through water. For instance, Ma et al. [92] used metagenomic data sets to investigate the relationship between ARGs, hosts, and environments. Figure 2 summarizes the main methods for detecting AR in aqueous environments, showing the advantages and disadvantages of the approaches.

## 5. Rapid Detection of AR—Contribution of Biosensors

**Figure 3:**
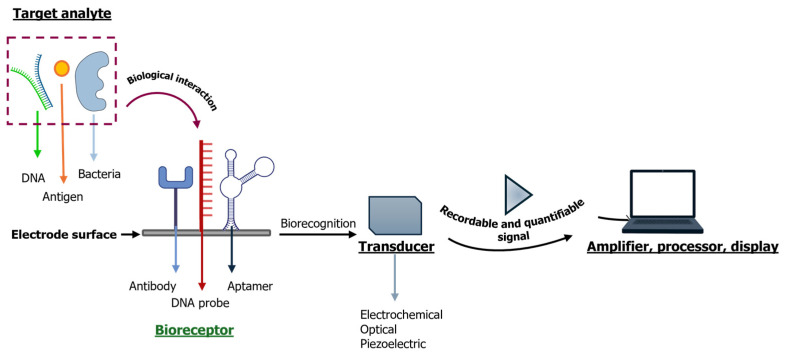
General biosensor components. The target analyte (such as DNAs, antigens, bacteria) binds with the biological recognition element (bioreceptor, such as antibodies, DNA probes and aptamers), generating a biological interaction. Then the electrochemical, optical or piezoelectric transducer converts the signal into a recordable and quantifiable signal. The signal is amplified, processed and displayed on a computer monitor.

Ideally, the goal would be to detect AR and determine antimicrobial susceptibility directly from the samples within about 30 min [93]. Biosensors detect bacteria, specific genes or proteins and drugs with high specificity, a short response time, and practical applications [94]. The use of these tools to identify the presence of AR could be a beneficial approach to monitor resistant bacteria in aqueous environments. In recent years, the development of biosensor-based approaches has shown great potential in speeding up the detection of AR and antibiotic resistance genes (ARGs), offering promising advantages for both clinical practice and field diagnostics. A biosensor typically includes a biological component, called a bioreceptor, that specifically binds to the target molecule. This interaction triggers a detectable signal, which can be a change in mass, fluorescence, electric charge, or even refractive index. The specificity of the bioreceptor is crucial, and it can vary depending on the sensor: for example, antibodies are used in immunosensors, DNA probes in genosensors, and aptamers (short DNA or RNA strands) in aptasensors. The signal produced by this interaction is then captured by a transducer, which converts it into measurable data. Depending on the type of signal conversion, biosensors can be optical (based on light changes), piezoelectric (using material deformation to sense interactions), electrical (based on changes in electrical properties), or electrochemical (where the signal is based on electron transfer processes) (Figure 3).

A growing number of scientific investigations are now exploring the use of biosensors with electrochemical transducers for the detection of AR. These systems leverage various techniques, including voltammetry, which involves the application of a constant/varying potential to the surface of an electrode and recording a Faradaic current that flows in a working electrode to determine the presence and provide a semi-quantification of the target analyte; impedance spectroscopy, which applies a range of frequencies to evaluate parameters like capacitance and electron transfer resistance; and amperometry, where the oxidation or reduction of analyte molecules is directly translated into a measurable current. Another commonly used method is conductivity-based sensing, which relies on the application of different frequencies to assess changes in conductance [95,96]. Based on these techniques, ARGs can be detected by short oligonucleotide sequences immobilized onto transducer surfaces: methylene blue-labelled hairpin capture probes were used to detect the *mecA* gene in methicillin-resistant *S. aureus*, for example [97]. Electrochemical biosensors present several advantages, including rapid detection times, cost-effectiveness, and high specificity and sensitivity, as well as suitability for point-of-care applications in water environments. Another practical benefit is the minimal sample volume required for analysis [94]. While a preliminary DNA extraction step from bacterial cells is typically necessary, the actual gene detection process using biosensors is relatively fast and often completed within two hours [94]. When ARGs are the target, electrochemical genosensors combined with DNA hybridization have shown great promise. This approach involves immobilizing thiol-modified single-stranded DNA (ssDNA) probes onto the gold working electrode (WE) surface of screen-printed gold electrodes (SPAuE) through spontaneous covalent bonding. The complementary target sequence then hybridizes with the probe, enabling detection. Using this strategy, specific ARGs have been successfully identified in under one hour [98]. Similar techniques with comparable detection times have been applied in other studies for multiplex ARG identification [99]. In recent years, electrochemical measurements have been combined with other molecular techniques, including isothermal amplification reactions. In a recent study [100], a recombinase polymerase amplification reaction was employed to preserve the activity of nucleotides labelled with horseradish peroxidase, and the amplification product was subsequently measured using an electrochemical biosensor. The horseradish peroxidase as the redox enzyme favoured the biosensor readout. The results were promising; in fact, *E. coli* carrying the oxacillin resistance gene was detected at levels as low as 319 CFU/mL and 10.6 aM of plasmid DNA within one hour. However, the authors noted that further optimization is required to reduce the overall assay time. The advantage of this technique lies in the simultaneous amplification and target capture directly on the electrode surface, eliminating the need for prior DNA extraction and the generation of single-stranded DNA for hybridization-based detection.

Electrochemical Impedance Spectroscopy (EIS) is closely associated with the family of electrochemical biosensors. EIS involves the application of a sinusoidal excitation signal to the system and the measurement of the resulting current, voltage, or potential response as a function of frequency. This technique has a wide range of applications, including food and water quality analysis and, notably, the detection of pharmaceutical compounds and agents associated with AR. The presence of pharmaceutically active materials in the environment is critical in the spread of AR. Often, these substances are present at extremely low concentrations, making their detection challenging with conventional techniques. EIS offers an exceptionally low limit of detection, capable of identifying target analytes at concentrations as low a 10^−12^ M. Applications were explored for the detection of various drug targets, including oxytetracycline, tetracycline, tobramycin, chloramphenicol, and sulphamethoxazole, as well as pathogenic bacteria such as *Salmonella* species in food matrices, with detection limits as low as 3 CFU/mL. The use of small-amplitude excitation signals and low frequencies makes EIS particularly suitable for practical applications. This allows EIS to function as a steady-state, non-destructive analytical technique, which is advantageous for real-time and in situ monitoring [101].

Another class of biosensors relevant for the detection of AR in various environments includes nanobalances, such as Quartz Crystal Microbalance (QCM). It allows piezoelectric measurements in real-time by recording resonant frequency with mass changes on the transducer surface on which specific capture probes can be immobilized. Moreover, with this sensor, testing antibiotic effectiveness or ineffectiveness against bacteria that grew or did not grow on the transducer surface was possible [94]. Also, electrophysical biosensors play a considerable role. Flow cytometry (FCM) is an advanced technique which exploits light scattering and fluorescence phenomena to analyze cellular populations and subpopulations [102]. This method enables the characterization of up to 20 different bacterial properties within a few minutes. The response of bacteria to antibiotics can be evaluated using FCM alone or in combination with Matrix-Assisted Laser Desorption/Ionization Time-of-Flight Mass Spectrometry (MALDI-TOF MS). Within less than two hours, several classes of antibiotics were tested on Gram-positive and Gram-negative clinical bacterial strains, and microbial mortality levels were assessed without the need for bacterial growth on culture media. Moreover, by using fluorescent dyes, FCM can differentiate between viable cells, cells in an intermediate physiological state, and dead cells within a microbial population [103].

Both EIS and FCM analyses typically require relatively large sample volumes to produce a detectable signal variation, for example, impedance. To overcome this limitation, photonic biosensors offer an effective alternative, providing results from small sample volumes within a short analysis time (approximately one hour). Photonic biosensors use light interaction with micro- or nanoscale structures to detect specific analytes. The analyte interaction induces changes in the refractive index, optical phase, or light intensity, enabling highly sensitive detection. A photonic approach was proposed to evaluate the effectiveness of antibiotics on bacterial cells [104]. The authors used nanophotonic traps to realize an array based on photonic crystal cavities where bacteria were trapped, and, after antibiotic exposure, changes in optical properties were monitored. Additionally, the medium’s impedance was used to evaluate the metabolic activity of bacteria in contact with the drugs. However, such biosensors are often complex to fabricate, challenging to miniaturize, and require expert personnel for data interpretation. To address these limitations, photonic principles were integrated with microfluidic systems, realizing “lab-on-a-chip” platforms, low-power devices capable of providing results directly on a single chip. Microfluidics refers to the manipulation of small volumes of fluids at the microscale within channels of micrometer dimensions. When integrated with electrodes, these systems can apply small voltages to generate large, localized electric fields, enabling precise control of biochemical processes [105].

The coupling of microfluidics with impedance spectroscopy has led to the development of “organs-on-chip”, miniaturized platforms that mimic the physiology of human organs by replicating tissue structures, fluid flow, biochemical gradients (such as pH), and the microenvironment where cellular biochemical reactions occur [106]. This innovation holds significant promise for future studies of interactions between human cells and AR pathogenic bacteria. Furthermore, a study by di Toma et al. [107] proposed an on-chip platform which combines microfluidics with dielectrophoresis (DEP). DEP refers to the movement of polarizable particles in a non-uniform electric field, unlike electrophoresis which acts on charged particles in a uniform electric field. This approach enabled the creation of a microfluidic channel integrated with electrodes, where bacteria were exposed to antibiotics for approximately one hour. Subsequently, the system was able to differentiate between live and dead cells in just 5 s, achieving a classification efficiency greater than 98%.

There are several potential applications of biosensors. These include the detection of AR bacteria, the evaluation of antibiotic efficacy on microorganisms, and the monitoring of pharmaceutical residues in the environment. To be applied in real-world settings, an ideal biosensor should offer a short analysis time; high selectivity, sensitivity, and precision; low operational costs; and good reproducibility, compatibility, and portability [108]. Thanks to these attributes, biosensors are emerging as a viable alternative to traditional culture-based and molecular diagnostic methods [109]. Prompt and accurate identification of AR infections is fundamental for effective treatment. Biosensors not only guide the appropriate selection of antibiotics to combat infections by detecting specific targets responsible for AR but also support public health efforts by tracking the emergence and dissemination of resistance. Traditional diagnostic procedures for bacterial infections often take several days to yield comprehensive results. However, in cases involving critically ill patients, such delays are unacceptable, as therapeutic decisions must often be made within the first 6 to 12 h of infection to improve outcomes and reduce mortality [93,110]. The development of rapid, sensitive, and precise diagnostic technologies is essential for timely response and global health protection [111]. The economic burden associated with hospitalizations and patient treatments remains high, and by 2050 a global financial crisis with a loss of EUR 3.4 trillion in the global economy is expected [112,113]. Routine diagnostic analyses are often costly, both in terms of materials and labour. Therefore, the development of low-cost analytical methods could significantly contribute to improving overall healthcare sustainability. Using compact and portable biosensor devices enables on-site testing at the point of sample collection and eliminates the need for sample transportation and complex laboratory procedures. Moreover, since antibiotic treatment decisions are directly linked to patient survival, it is crucial to ensure robust reproducibility of test results to minimize the risk of false positives and the subsequent administration of inappropriate therapies. Once fully optimized, such systems may even enable direct detection of bacteria, drugs and ARGs in complex, unpurified, multi-microbial contaminated water samples. To better identify the most suitable biosensing technique, Table 4 summarizes the main characteristics discussed throughout the text.

## 6. Conclusions and Outlook

The problem of AR is serious and alarming all over the world and in many areas, especially in the health and food sectors. Mismanagement of the threat, drug abuse, and external climatic conditions are worsening the situation, so quick and effective actions are needed. Water is often the means for bacteria spread, so it is essential to evaluate the entire aqueous flow as one of the main factors responsible for horizontal gene transfer from farm to fork, causing hospitalization and economic losses.

The emergence of AR bacteria is closely linked with surface water pollution due to inadequate wastewater treatment that enables the survival of resistant bacteria and their ARGs. Polluted surface water acts as a vehicle for the dissemination of AR in food, animals and humans through direct or indirect contact. Water-mediated spreading and persistence of AR pathogens are facilitated by human activities and environmental factors, such as global warming, imposing the need for continuous monitoring of livestock, agri-food, and aquaculture systems. Sanitary and control measures are more implemented in urban areas compared to agriculture and aquaculture areas, where wastewater flows into deep surface waters or bodies with larger volumes and may lead to the spread of resistant bacterial strains and their genes. Many pathogenic bacteria are capable of surviving water treatment and persist in the environment. In addition, non-pathogenic bacterial strains may acquire resistance under selective pressure due to the presence of antibiotic residues and various contaminants in aquatic environments, contributing to the spread of resistance.

Acting now is essential to avoid an AR pandemic: it is necessary to use alternative solutions to common antibiotics, and modern healthcare and environmental surveillance is required to identify, quickly and at low-cost, the presence of resistant bacteria. All professional figures should be involved, both in the agri-food and medical fields, in waste disposal and water treatment plants, to find a complete and effective line of action.

## Figures and Tables

**Table 1:** Examples of antibiotic resistance found in water systems affected by livestock production.

Sources	Country	Antibiotic—Antibiotic Class	Significance in Human Therapeutics *	Reference
Swine	North Carolina	Ampicillin—Penicillins	Critically important	[25]
		Chloramphenicol—Amphenicols	Highly important	
		Sulphamethoxazole—Sulphonamides	Highly important	
		Sulfisoxazole—Sulphonamides	Highly important	
		Streptomycin—Aminoglycosides	Critically important	[24]
		Tetracycline—Tetracyclines	Highly important	
Swine and poultry	China	Ampicillin—Penicillins	Critically important	[23]
		Chlortetracycline—Tetracyclines	Highly important	
		Gentamycin—Aminoglycosides	Critically important	
		Sulphamethoxazole/Trimethoprim—Sulphonamides	Highly important	
		Tetracycline—Tetracyclines	Highly important	
Livestock (generic)	Taiwan	Sulphamethoxazole/Trimethoprim—Sulphonamides	Highly important	[20]
		Tetracycline—Tetracyclines	Highly important	
Poultry	Bangladesh	Amoxicillin—Penicillins	Critically important	[28]
		Azithromycin—Macrolides	Critically important	
		Ciprofloxacin—Quinolones	Critically important	
		Erythromycin—Macrolides	Critically important	
		Gentamycin—Aminoglycosides	Critically important	
		Norfloxacin—Quinolones	Critically important	
		Streptomycin—Aminoglycosides	Critically important	
		Tetracycline—Tetracyclines	Highly important	

* Based on [4].

**Table 2:** Examples of antibiotic resistance detected in agricultural water waste.

Sources	Country	Antibiotic—Antibiotic Class	Significance in Human Therapeutics *	Reference
Manure, soil, vegetables and groundwater	China	Tetracycline—Tetracyclines	Highly important	[30]
		Sulphonamides	Highly important	
		Quinolones	Critically important	
Sediments fertilized by pig slurries	Italy	Oxytetracycline—Tetracyclines	Highly important	[31]
Overland flow after putting the slurry to arable land	United Kingdom	Oxytetracycline—Tetracyclines	Highly important	[32]
		Sulphachloropyridazine—Sulphonamides	Highly important	
Poultry litter-soil-water environment	United States	Monensin, salinomycin and narasin—Ionophores	Currently not used in humans	[33]
Swine manure	United States	Streptomycin—Aminoglycosides	Critically important	[35]
		Sulphisoxazole—Sulphonamides	Highly important	
		Tetracycline—Tetracyclines	Highly important	
Avian organic fertilizer	Brazil	Tetracycline—Tetracyclines	Highly important	[36]
		Gentamycin—Aminoglycosides	Critically important	
		Cefotaxime—Cephalosporins	Critically important	
		Nitrofurantoin—Nitrofurans	Important	
		Trimethoprim/sulfamethoxazole—Sulphonamides	Highly important	
		Ampicillin—Penicillins	Critically important	
Irrigation water, manure and soil	Nigeria	Quinolones	Critically important	[37]

* Based on [4].

**Table 3:** Examples of antibiotic resistance reported in aquaculture waters.

Sources	Country	Antibiotic—Antibiotic Class	Significance in Human Therapeutics *	Reference
Marine environments	United States	Ampicillin—Penicillins	Critically important	[50]
		Sulfadimethoxine—Sulphonamides	Highly important	
Salmon farm	Chile	Amoxicillin—Penicillins	Critically important	[47]
		Streptomycin—Aminoglycosides	Critically important	
		Tetracycline—Tetracyclines	Highly important	
		Trimethoprim/Sulfamethizole—Sulphonamides	Highly important	
Aquatic animals	China	Chloramphenicol—Amphenicols	Highly important	[49]
		Sulphonamide—Sulphonamides	Highly important	
		Tetracycline—Tetracyclines	Highly important	
Fish farms	Finland	Aminoglycoside—Aminoglycosides	Critically important	[48]
		Chloramphenicol—Amphenicols	Highly important	
		Sulfonamide—Sulphonamides	Highly important	
		Tetracycline—Tetracyclines	Highly important	
		Trimethoprim—Sulphonamides	Highly important	
Aquaculture farms	Singapore	Beta-lactams—Penicillins	Critically important	[46]
Farmed freshwater fish	Hong Kong	Beta-lactams—Penicillins	Critically important	[51]
		Carbapenemase-producers—Carbapenems	Critically important	

* Based on [4].

**Table 4:** Different types of biosensors for the monitoring and control of antibiotic resistance.

Biosensor Technique	Target Detection	Strengths	Limitations	Refs.
Electrochemical biosensors	ARGs	Fast detection (<2 h), cost-effective, highly specific and sensitive, suitable for point-of-care in water environments, requires minimal sample volume	Pre-analysis DNA extraction required; limited to specific target genes	[94,97,98,99]
Electrochemical biosensors + isothermal amplification	ARGs	Fast detection (<1 h), combined amplification and target capture directly on electrode surface; avoids need for DNA extraction and ssDNA generation	Not yet optimized; may be inactivated by thermal cycling	[100]
Electrochemical Impedance Spectroscopy (EIS)	Bacteria, drugs, pharmaceutical residues	Steady-state, non-destructive technique; label-free; real-time monitoring	Requires large sample volume; sensitivity depends on electrode surface preparation	[101]
Quartz Crystal Microbalance (QCM)	ARGs Bacteria Drug effect	Real-time measurement No sample damage	Sensitive to external disturbances	[94]
Impedance Flow Cytometry (FCM)	Phenotypic resistance in bacteria	Fast profiling (<2 h) of Gram+/Gram– strains; distinguishes viable, intermediate, and dead cells; no need for culture; can test multiple antibiotics simultaneously	Large sample volume; requires fluorescent labelling for detailed analysis	[102,103]
Photonic biosensors	Antibiotic effect on bacteria	Analysis time 1 h, use of photonic crystal cavities to trap cells and monitor metabolic/optical response; low sample volume	Complex fabrication; low portability; high instrumentation cost	[104]
Lab-on-Chip with Photonic Integration	Bacterial or cell response	Miniaturized, low-power device; integrates optics and microfluidics for single-chip results	Complexity in design and fluid control; limited scalability	[105]
Organ-on-Chip with Impedance Spectroscopy	Cell–drug/ bacteria interaction	Simulates human tissues and physiological conditions; replicates pH, flow, and biochemical environment	Complex design; primarily research-stage	[106]
Lab-on-Chip with Dielectrophoresis (DEP)	Cell viability under antibiotic stress	Integrated microfluidic channel with electrodes; distinguishes live/dead cells in ~5 s after 1 h antibiotic exposure; >98% accuracy	Requires precise electrode configuration; fabrication complexity	[107]

## Data Availability

The original contributions presented in the study are included in the article, further inquiries can be directed to the corresponding author.
